# Efficacy of autologous platelet-rich plasma on graft uptake in myringoplasty: a single-blinded randomized control trial

**DOI:** 10.1186/s43163-021-00190-x

**Published:** 2022-01-15

**Authors:** R. Vignesh, V. Nirmal Coumare, S. Gopalakrishnan, P. Karthikeyan

**Affiliations:** Department of Otorhinolaryngology and Head and Neck Surgery, Mahatma Gandhi Medical College and Research Institute, (Sri Balaji Vidyapeeth)), Pondicherry, India

**Keywords:** Platelet-rich plasma, Myringoplasty, Autologous, Graft uptake, Platelets

## Abstract

**Background and aim:**

Chronic otitis media is defined as a chronic inflammation of the middle ear cleft producing irreversible pathological changes. The myringoplasty aims at the reconstruction of the tympanic membrane using a graft material. Autologous platelet-rich plasma can be used along with the graft to have a successful outcome. The aim of the study was to assess the efficacy of autologous platelet-rich plasma on graft uptake in myringoplasty.

**Methods:**

This was a randomized controlled trial with a sample size of 76 patients. All patients were above 18 and below 55 years of age diagnosed with chronic suppurative otitis media and were planned for myringoplasty. The participants were randomly allotted to two groups by block randomization (block of 4). Intra-operatively, group I (*n*=38) received platelet-rich plasma–soaked gel foam and group II (*n*=38) was taken as the control group who received saline-soaked gel foam and examined by a blinded examiner at the end of 1st and 3rd months.

**Results:**

The mean air-bone gap reduction post-operatively in the platelet-rich plasma group was 8.68 ± 4.8 (*P* value 0.034) and 6.05 ± 4.05 in the control group. The improvement in *pure-tone average* in the platelet-rich plasma group (*P* = 0.009) is more than that in the control group. The graft uptake was higher among the platelet-rich plasma group than the control group both at 1st and 3rd months (*P* value 0.049) which were statistically significant.

**Conclusion:**

The present study concludes that the usage of platelet-rich plasma in the conventional myringoplasty technique has improved the success rate of graft uptake and reduced the graft migration.

**Trial registration:**

Clinical Trials Registry-India (ICMR-NIMS) CTRI/2020/04/024416. Date of registration: 01/04/2020. Date of enrolment of the first participant to the trial: 06/04/2020. URL of the trial registry: http://www.ctri.nic.in.

**Highlights:**

- Usage of autologous platelet-rich plasma (PRP) in conventional myringoplasty in underlay technique.
- Assessment of graft uptake, percentage of perforation closure, and the audiological outcome.
- Significant mean reduction of ABG in the PRP group.
- Significant improvement in PTA average in the PRP group.
- Graft uptake and percentage of perforation closure were higher in the PRP group and the control group.
- PRP is also beneficial in revision cases.

## Background/introduction

Chronic otitis media (COM) is one of the major otological problems causing hearing loss in developing countries. It is defined as the “persistent inflammation of the middle ear cleft” [1]. Apart from the medical measures, myringoplasty remains the mainstay of surgical treatment for COM for the repair of tympanic membrane perforations. The history of this surgery was dated back to the sixteenth century [2]. The myringoplasty aims at the reconstruction of the tympanic membrane using a graft material [3]. The success rate of myringoplasty has been ranged from 70 and 80% [4]. There are multiple factors responsible for the failure of the procedure like upper airway infection, type of the procedure, grafts, and trapped epithelial cell [5]. For repairing the tympanic membrane, there is a pursuit for new biomaterials or biological tissues which are cost effective and safe, which tends to have better outcome.

Autologous serum and autologous platelet-rich plasma have been used along with graft to have better successful outcomes. Since the introduction of platelet-rich plasma in the 1980s and 1990s, it has been used in many surgical procedures [6]. Platelet-rich plasma (PRP) contains 95% platelets, 4% RBCs, and 1% WBC. Some of the other factors from PRP are platelet-derived growth factor, TGF-β1, vascular endothelial growth factor, insulin-like growth factor, interleukin, etc. These factors play an important role in chemotaxis, mitogenesis, metabolism, differentiation, etc. [6]. Being an autologous product, PRP is safer as there is no chance of disease transmission and it does not have a mutagenic effect [7]. The preparation and extraction of PRP is easy and quick. There are very few studies evaluating the role of platelet-rich plasma in patients with chronic suppurative otitis media. Hence, this study was planned, to find out the efficacy of platelet-rich plasma on graft uptake in patients undergoing myringoplasty.

## Methods

This study was a randomized controlled study (registration number CTRI/2020/04/024416) conducted in Mahatma Gandhi Medical College and Hospital, Pondicherry, India, from April 2020 to February 2021 after the approval of the Institutional medical ethical committee. The study population included 76 patients above 18 and below 55 years of age with a clinical diagnosis of chronic otitis media and undergoing myringoplasty in the Department of Otorhinolaryngology during this period. Patients with active infection, attico-antral disease, chronic granulomatous disease and tumours of the ear, and co-morbidities like uncontrolled diabetes; patients with contraindications to general anaesthesia; and patients who smoked were excluded from this study. Prior to surgery, patients were randomly allocated into 2 groups by block randomization (block of 4):

- Group I: Platelet-rich plasma group (*n*=38)
- Group II: Control group (*n*=38)

In group I patients, 10 mL of blood was collected in the morning of the surgery at the time of insertion of an IV cannula, 3 h prior to surgery. The collected sample was sent to the blood bank for the preparation of platelet-rich plasma (PRP), which was then collected and brought to the operation theatre.

Patients in both groups underwent myringoplasty by using the underlay technique and temporalis fascia graft. In the study group, after placing the graft and repositioning the tympanomeatal flap, gel foam soaked in platelet-rich plasma was kept over the sealed perforation. In the control group, gel foam soaked in saline was kept over the sealed perforation, after repositioning the flap. Both groups of patients received antibiotics, as per the routine protocol followed by the department. Following the surgery, graft uptake was assessed by an otoscope at 1st and 3rd months post-surgery by a single-blinded observer. There were three outcome variables of the study: (a) to assess the graft uptake—success or failure, (b) to assess the percentage of perforation closure, and (c) to assess the audiological outcome.

### Statistical analysis

**Fig. 1:**
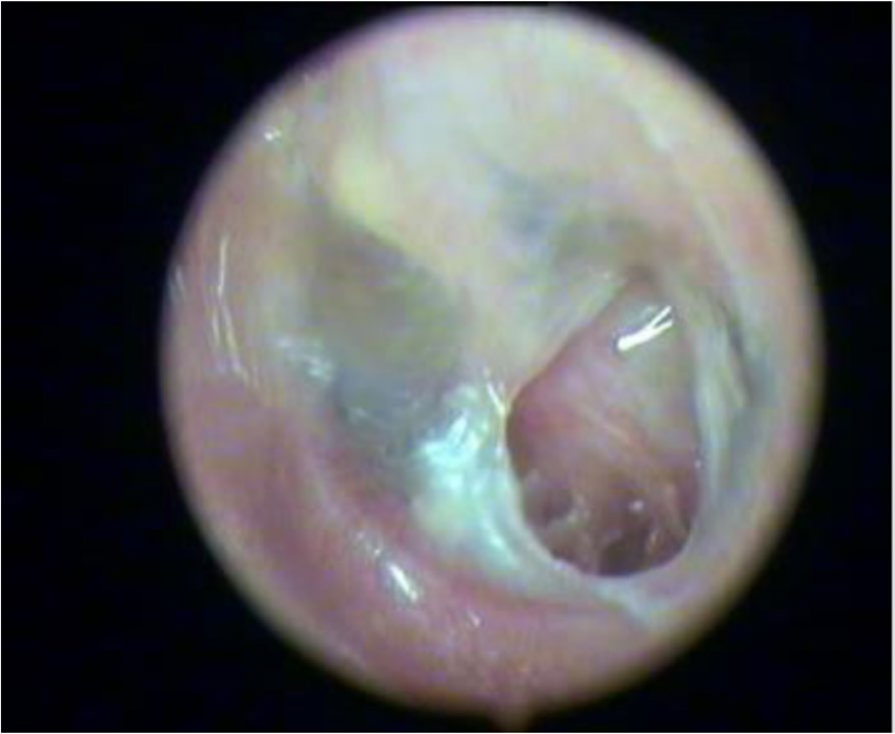
Moderate central perforation

**Fig. 2:**
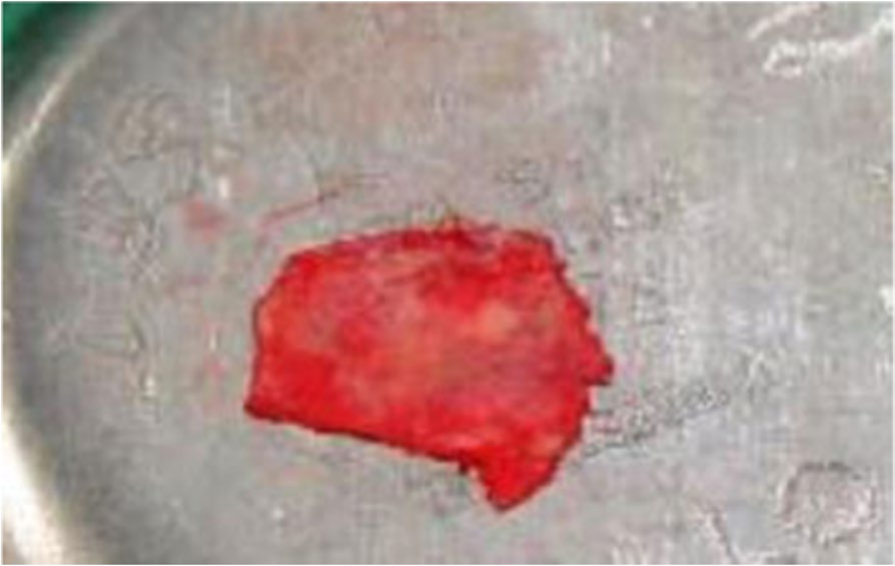
Temporalis fascia graft

**Fig. 3:**
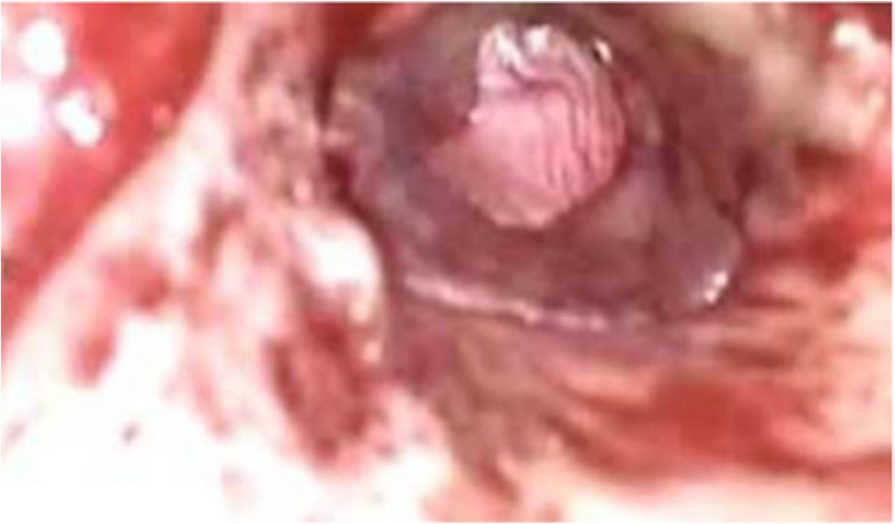
Underlay myringoplasty (intra-operative view)

**Fig. 4:**
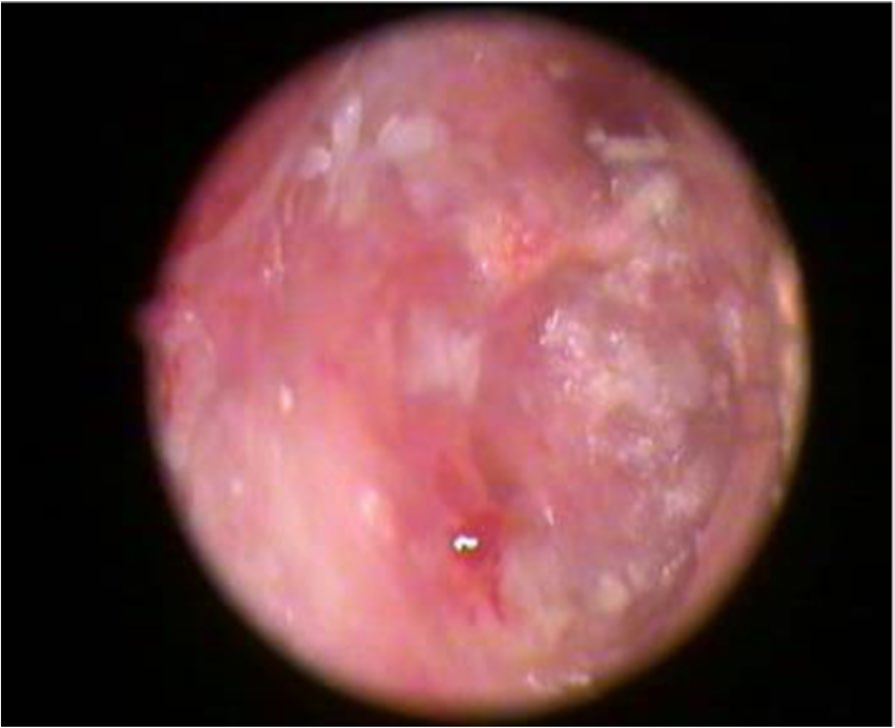
Graft uptake at 1st month

**Fig. 5:**
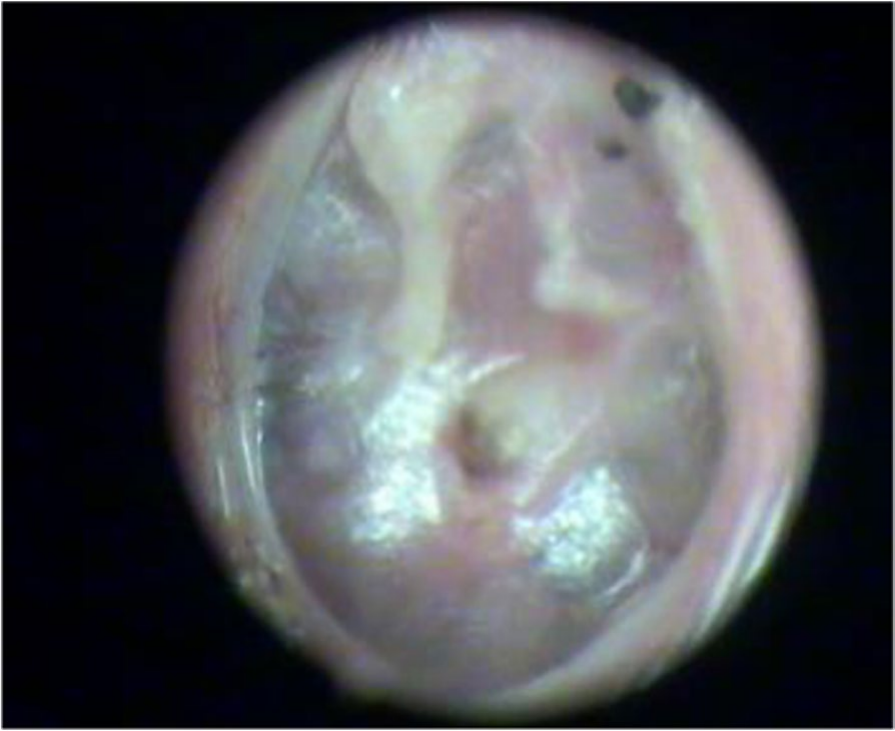
Graft uptake at 3rd month

The collected data was checked for completeness before entering into a Microsoft Excel spreadsheet. The validation of the data was checked at regular intervals. Data analysis was performed with an intention to treat approach using Statistical Package for Social Sciences (SPSS IBM) 19. The quantitative data was expressed in proportions. The Kruskal-Wallis test and Pearson chi-squared test were applied to find the association, and *P* value less than 0.05 will be considered significant (Figs. 1, 2, 3, 4, and 5).

## Results

**Fig. 6:**
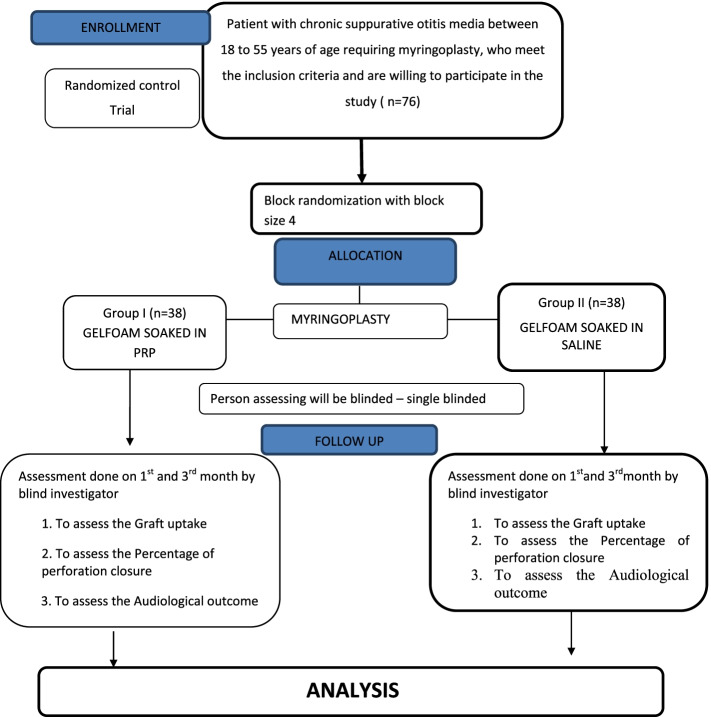
The CONSORT

The CONSORT (Consolidated Standards of Reporting Trials) for our study is depicted in Fig. 6.

The mean age of the study participants in the control group is 30.84 ± 8.86, and the mean age of the study participants in the PRP group is 29 ± 8.57. Most of the study participants (39.5%) in both groups are 21–30 years. In the PRP group, the majority (55.3%) of the study participants were males, and in the control group, 50% were males and females each. In the PRP group, 42.1% presented with hard of hearing as a symptom, and in the control group, 55.3% presented with hard of hearing. In the PRP group, 26.3% had ear discharge, and 18.4% had ear discharge in the control group. Among those who had ear discharge, it was present nearly 6–8 weeks before the surgery. At the time of surgery, all the participants had dry ear. 47.4% in the PRP group had ear ache, and 28.9% had ear ache in the control group. 55.3% had tinnitus in the PRP group, and 47.4% showed tinnitus in the control group. Fifty percent of the study participant’s blood group in the PRP group was O followed by B positive (28.9%), A positive (13.2%), and B negative (7.9%). The majority of the study participant’s blood group in the control group was O followed by B positive (31.6%), A positive (15.8%), and B negative (5.3%).

The majority of the study participants (63.2%) in the PRP group had a moderate central type of perforation followed by subtotal (28.9%) and small central (7.9%) type of perforation. Similarly, the majority of the study participants (52.6%) in the control group had a moderate central type of perforation followed by subtotal (31.6%) and (15.8%) small central type of perforation. The majority showed cellular changes in X-ray mastoid in both groups.

There was a significant reduction in the mean ABG after the operation in both groups. The mean ABG difference in the PRP group was 8.68 ± 4.8, and the mean ABG difference in the control group was 6.05 ± 4.05 with *P* value 0.034 which was statistically significant.

The improvement in *PTA* (average) in the PRP group is more than that in the control group. In the PRP group, 14 participants had a *PTA* difference (from pre-operative to 3rd month post-operative periods) of more than 10dB, whereas in the control group only 6 had more than 10-dB difference with *P* value 0.009 which was statistically significant.

Nearly 97.4% had graft uptake at 1st month in the PRP group, which is higher than the control group, which had only 89.5% graft uptake. Similarly, at 3 months, the graft uptake was higher among the PRP group than the control group with *P* value 0.037 which was statistically significant.

**Fig. 7:**
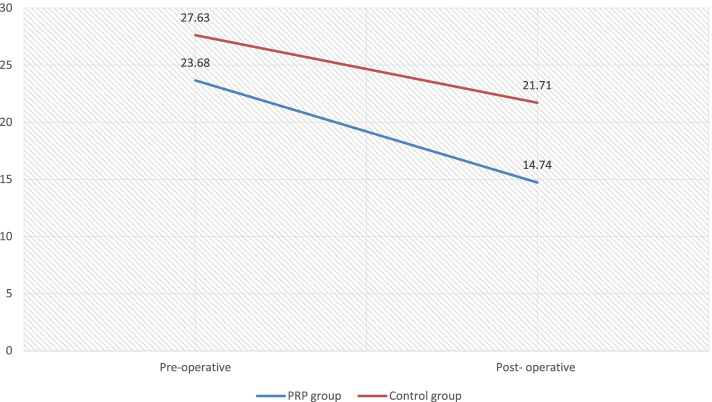
Graphical representation of ABG closure in the pre-operative and post-operative period among the groups

**Fig. 8:**
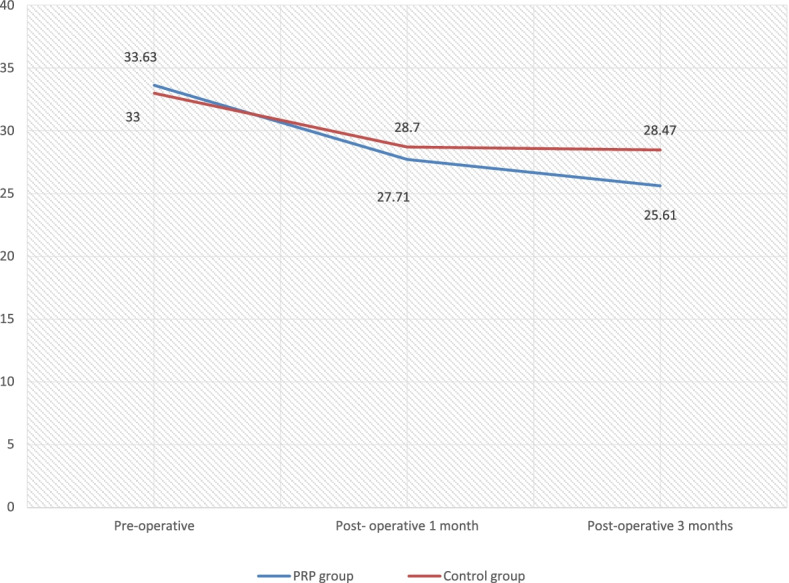
Graphical representation of PTA in the pre-operative and post-operative periods in both groups

**Fig. 9:**
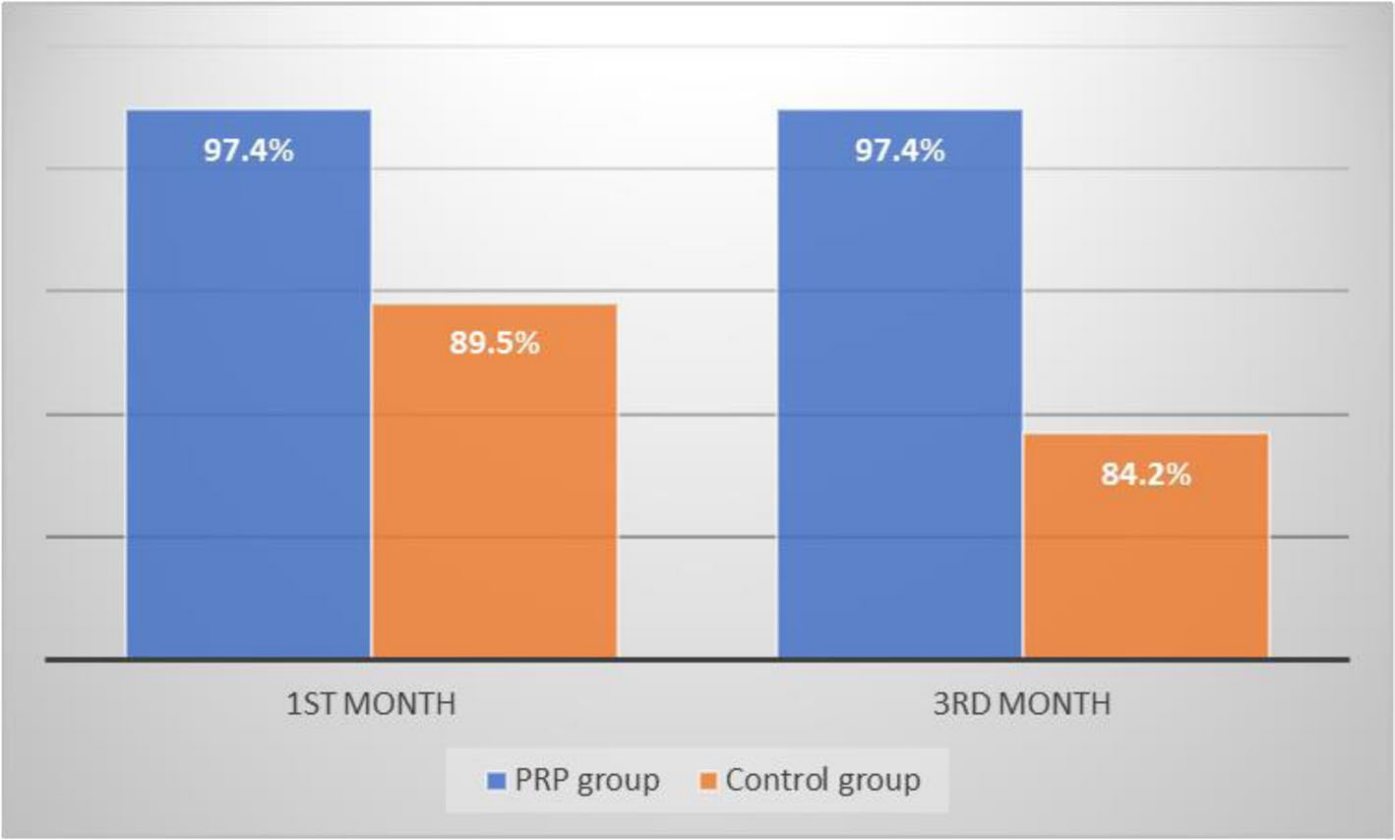
Graphical representation of graft uptake at 1st and 3rd months among both groups

**Fig. 10:**
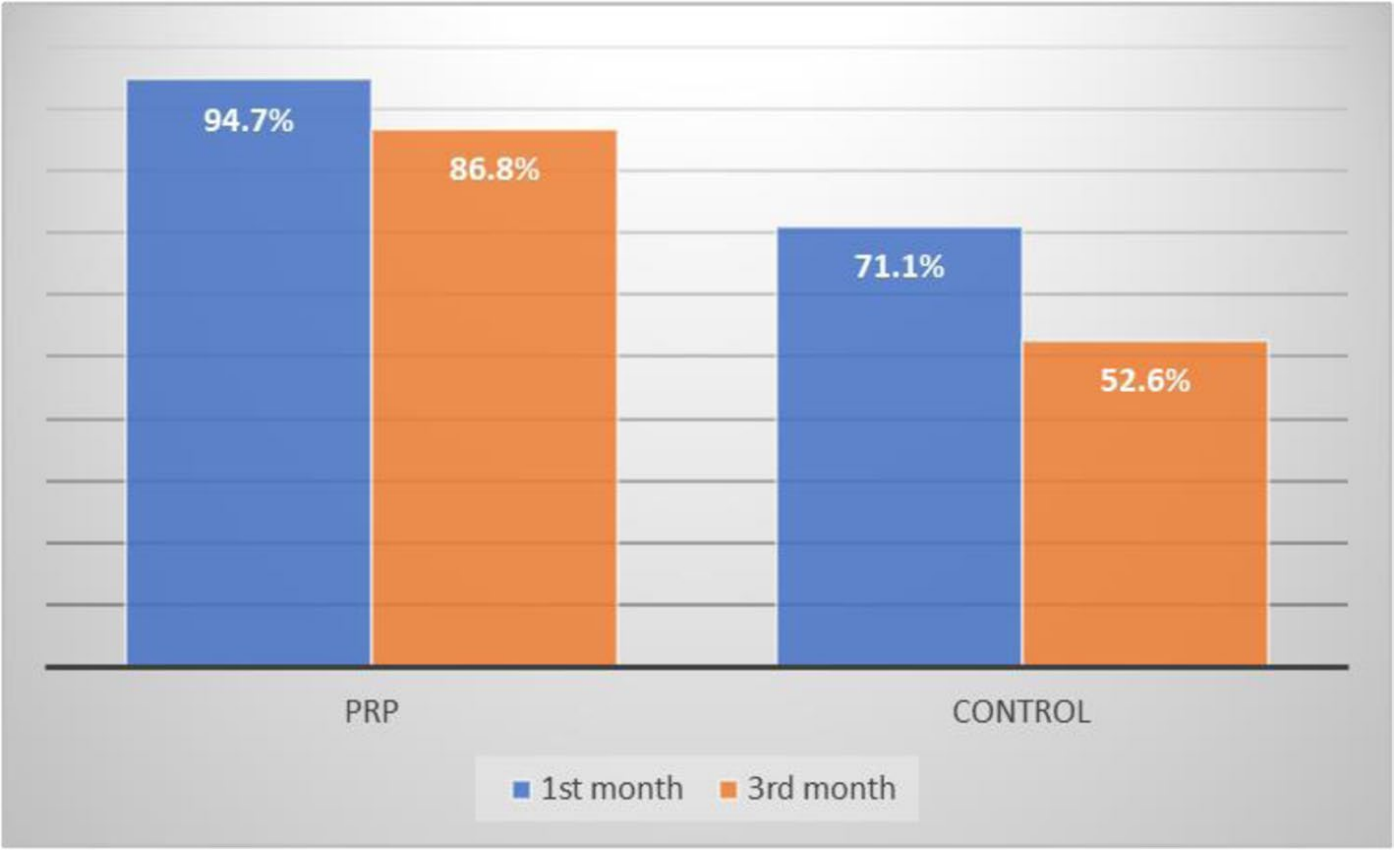
Graphical representation of complete closure at 1st and 3rd months among both the groups

One hundred percent closure at the 1st month of the post-operative period was seen in 96.4% and 71.1% in the PRP and control group respectively. Similarly, at the 3rd month post-operative period, 86.8% and 52.6% had 100% closure in the PRP and control groups respectively with *P* value 0.034 which was statically significant. In both groups, the 100% closure failure was seen maximum in subtotal perforations. The closure results were good in small perforation followed by moderate perforation (Figs. 7, 8, 9, and 10).

### Revision cases

There were totally 8 revision cases and all the revision cases had undergone cortical mastoidectomy along with myringoplasty. There is a reduction in *PTA* in the revision cases among both groups, but it is not statistically significant. The maximum *PTA* difference in the PRP group was 11dB (25%), whereas it is 6dB in the control group (25%). All the revision cases in the PRP group had 100% graft uptake; however, only 75% had graft uptake in the control group at 1st and 3rd months. All 4 revision cases in the PRP group had 100% closure at 1st and 3rd months in the post-operative period, whereas in the control group only 50% had 100% closure.

## Discussion

Myringoplasty is the main surgical treatment used for the tympanic membrane reconstruction using grafts in chronic otitis media–mucosal type-inactive. Platelet-rich plasma, an autologous serum, is rich in proteins like fibrin, fibronectin, vitronectin, and various growth factors [8]. The special proteins help in clot formation and thereby enhance healing. It is superior to fibrin glue which does not contain growth factor. With this background, the present study was conducted with the aim to assess the efficacy of autologous platelet-rich plasma on graft uptake in myringoplasty which yield significant mean reduction in the air-bone gap, improvement in pure-tone average, graft uptake, and percentage of closure after surgery which was higher in the PRP group when compared to the control group.

The mean age of the study participants in the control group is 30.84 ± 8.86, and the mean age of the study participants in the PRP group is 29 ± 8.57. Most of the study participants (39.5%) in both groups is 21–30 years with majority male in PRP and equal in the control group. Similarly, studies done by Fawzy et al. and Singh et al. showed the mean age of the study participants was around 28.5 ± 5.9 years [9, 10]. The present study showed in both groups together, 37 (48.7%) had hard of hearing and 17 (22.3%) had ear discharge. It should be noted that among those who had ear discharge, it was present nearly 6–8 weeks before the surgery. At the time of surgery, all the participants had dry ear.

The present study showed a significant reduction in ABG after surgery. The mean reduction of ABG in the PRP group was 8.68 ± 4.8, and the mean ABG reduction in the control group was 6.05 ± 4.05. The ABG reduction was higher among the PRP group. This is in accordance with a study done by Ersozlu T et al. which showed that the mean ABG difference in the PRP group is 10.3 ± 6.74 and in the control group was 7.23 ± 6.72. A study done by Ebrahim et al. and Shanmugam et al. showed that the mean difference in the air-bone gap in those with PRP is 13.75 ± 5.59, which is slightly higher than the present study [11, 12].

The present study showed improvement in *PTA* (pure-tone average) after the procedure; however, the improvement in the PRP group is more than that in the control group. The mean *PTA* improvement in the PRP group during the pre-operative and 3-month post-operative period is 33.63 ± 2.85 and 27.71 ± 4.22 respectively whereas in the control group it was 33 ± 3.78 and 28.7 ± 4.5 in the pre- and post-operative period respectively. This is in accordance with studies done by Fawzy et al., El-Anwar et al., and Hosam et al. which showed a reduction in *PTA* in the PRP group from 23 ± 4.70 in the pre-operative period and 16.50 ±6.51 in the post-operative period [13–15].

The present study showed that nearly 97.4% (37) had graft uptake at 1st month in the PRP group, which is higher than the control group, which had only 89.5% graft uptake. Similarly, at 3 months, the graft uptake was higher among the PRP group than the control group which is statistically significant. The result of the present study on graft uptake is in accordance with the study done by Yadav SPS et al. which showed that after the 3-month period the graft uptake was 95% and 85% in the PRP and control groups respectively. The studies by El-Anwar et al. and Fawzy et al. showed a much lower success rate of 84% than the present study [13, 14]. The present study thus in accordance with other studies showed that the addition of platelet-rich plasma resulted in higher graft uptake than the graft alone group and revealed that PRP would be important in the success rate of the myringoplasty procedure. The reason for the graft failure could be due to recurrent upper respiratory tract infection, Eustachian tube dysfunction, and poor compliance of instructions to be followed in the post-operative period including general hygiene by the patient.

The present study showed that the percentage of closure at 1st month and 3rd month was higher among the PRP group than the control group which was statistically significant. Near complete closure at the 1st month of the post-operative period was seen in 96.4% and 71.1% in the PRP and control group respectively. Similarly, at the 3rd month post-operative period, 86.8% and 52.6% had 100% closure in the PRP and control groups respectively. A study done by Sankaranarayanan G et al. stated that the closure in the PRP group was 72% whereas in the control group it was only 40% at the end of the 1st month. At the end of the 3rd month, the closure was 96% and 80% in the PRP group and the control group respectively, which matches the present study findings [1]. A study steered by Fouad et al. also proved the successful closure of the tympanic membrane in the PRP group was 85.7%, higher than that in the pure myringoplasty group [16]. Shiomi et al. stated that irrespective of the size of the perforation, addition of platelet-rich plasma to the conventional myringoplasty technique has significantly increased the success rate of graft uptake [17].

The revision cases also had improvement in *PTA* higher in the PRP group than the control group. Since the sample size in the revision case is small, the observed improvement in *PTA* is clinically significant even though it is not drawing any statistical significance. The study also reported that all the revision cases in the PRP group had 100% (4) graft uptake; however, only 75% (3) had graft uptake in the control group at 1st and 3rd months. All 4 revision cases in the PRP group had 100% closure at 1st and 3rd months in the post-operative period, whereas in the revision cases in the control group only 50% had complete closure.

The strength of this study is that it emphasizes the effectiveness of PRP in revision cases that yields good results and is the 1st study in South India to report on revision cases. It was examined by a single-blinded examiner for all the cases and documented. Since PRP is autologous, it is easy and safe to prepare. All 76 cases were operated by the same surgeon with a single technique to avoid bias and variation in results.

The limitations of our present study are this is a single-centre study, this has a smaller sample size, quantity and quality of PRP could not be standardized for all samples, and also because of the COVID-19 pandemic time, it was difficult to follow up.

## Conclusions

The present study concludes that the usage of platelet-rich plasma in the conventional myringoplasty technique has improved the success rate of graft uptake and reduced the graft migration. It has been shown to accelerate the closure of tympanic membranes. The addition of PRP has also revealed greater audiological outcome in terms of air-bone gap closure, in the pure-tone audiogram. Platelet-rich plasma, an autologous serum which is rich in proteins and growth factors, helps and promotes healing with no noticeable side effects in our study. Moreover, addition of PRP has not altered the cost of the procedure significantly. Though various literature signifies the usage and effectiveness of PRP in different fields of medicine and regenerative science, it lacks the standardization of method of preparation, quality and quantity of PRP used, concentration of platelets and WBC in PRP, and preparation and method of usage. Hence, further studies and research in PRP and its by-products are required for future use.

## Data Availability

The datasets used and/or analysed during the current study are available from the corresponding author on reasonable request.
